# Patients’ experiences and wellbeing after injury: A focus group study

**DOI:** 10.1371/journal.pone.0245198

**Published:** 2021-01-07

**Authors:** Eva Visser, Brenda Leontine Den Oudsten, Marjan Johanna Traa, Taco Gosens, Jolanda De Vries

**Affiliations:** 1 Trauma TopCare, ETZ Hospital (Elisabeth-TweeSteden Ziekenhuis), Tilburg, The Netherlands; 2 Center of Research on Psychological and Somatic Disorders, Department of Medical and Clinical Psychology, Tilburg University, Tilburg, The Netherlands; 3 Department of Medical Psychology; ETZ Hospital (Elisabeth-TweeSteden Ziekenhuis), Tilburg, The Netherlands; 4 Department of Orthopaedics, ETZ Hospital (Elisabeth-TweeSteden Ziekenhuis), Tilburg, The Netherlands; University of Birmingham, UNITED KINGDOM

## Abstract

**Background:**

Injury can have physical, psychological and social consequences. It is unclear which factors have an impact on patients’ wellbeing after injury. This study aimed to explore, using focus groups, patients’ experiences and wellbeing after injury and which factors, impede or facilitate patients’ wellbeing.

**Methods:**

Trauma patients, treated in the shock room of the Elisabeth-TweeSteden Hospital, the Netherlands, participated in focus groups. Purposive sampling was used. Exclusion criteria were younger than 18 years old, severe traumatic brain injury, dementia, and insufficient knowledge of the Dutch language. The interviews were recorded, transcribed verbatim, and analyzed using coding technique open, axial, and selective coding, based on phenomenological approach.

**Results:**

Six focus groups (3 to 7 participants) were held before data saturation was reached. In total, 134 patients were invited, 28 (21%) agreed to participate (Median age: 59.5; min. 18 –max. 84). Main reasons to decline were fear that the discussion would be too confronting or patients experienced no problems regarding the trauma or treatment. Participants experienced difficulties on physical (no recovery to pre-trauma level), psychological (fear of dying or for permanent limitations, symptoms of posttraumatic stress disorder, cognitive dysfunction), social (impact on relatives and social support) wellbeing. These are impeding factors for recovery. However, good communication, especially clarity about the injury and expectations concerning recovery and future perspectives could help patients in surrendering to care. Patients felt less helpless when they knew what to expect.

**Conclusions:**

This is the first study that explored patients’ experiences and wellbeing after injury. Patients reported that their injury had an impact on their physical, psychological, and social wellbeing up to 12 months after injury. Professionals with the knowledge of consequences after injury could improve their anticipation on patients’ need.

## Introduction

In 2017, mortality rates from injury were the highest in Dutch persons younger than 35 years of age compared to other ages [1]. Due to trauma registration and implementation of specialized trauma care, the quality of trauma care improved and survivorship increased [1–6]. Nevertheless, patients who were less satisfied with general health and recovery after injury needed more medical care, they had a longer hospital stay, and they visited the hospital more often [7]. This resulted in an increase in costs of care. In the Netherlands, the total costs of injuries were €3.5 billion annually [6,8].

After experiencing a single traumatic event (e.g., fall or car accident), survivors will go through a process of medical treatment and rehabilitation: from the ambulance or trauma helicopter to the shock room, possible hospital stay, and finally rehabilitation [9]. The shock room is situated at the emergency department and, for severely injured patients, it is the interface between prehospital management and inpatient care [10]. Adverse physical (e.g., problems on wound repair and pain) [11–13], psychological [14,15], and social (e.g., broken marriages and difficulties in resumption to work) [16,17] outcomes may occur after injury. Patients can experience anxiety [18], depressive symptoms [18,19], acute stress disorder (ASD) [20], and posttraumatic stress disorder (PTSD) [14,18,21,22] after injury. These consequences can arise almost directly after injury or months or years later [23–25]. Even though they are often not recognized, they can have an impact on patients’ wellbeing. Yet, it is unclear which factors have an impact on patients’ experiences and wellbeing after injury, treatment and recovery. For that reason, qualitative research is needed to evaluate patients’ experiences after injury and which factors impede or facilitate patients’ wellbeing.

Although patients’ perspectives after injury have previously been explored, they evaluated one type of injury (e.g., traumatic brain injury (TBI) or burn injuries) [26,27] or one type of trauma mechanism (e.g., motor vehicle accident) [28,29]. Therefore, results cannot be generalized to the entire trauma population. Research is focused on recovery from different types of injury (e.g., multi trauma, spinal cord injury, and TBI) [29] will provide a broader overview than currently available.

To our knowledge, no focus group study was previously conducted that focused on a process of trauma care (i.e., treatment short after injury, in the shock room and hospital, and rehabilitation) and patients’ wellbeing [30,31]. Therefore, this study aimed to explore patients’ experiences and wellbeing after injury, treatment, and rehabilitation. Moreover, factors that impede or facilitate patients’ wellbeing were evaluated.

## Material and methods

### Study design

A focus group study design was used to evaluate the aims of this study. Focus groups, a commonly used method of qualitative research [32,33], were held, because they facilitate an in-depth exploration of a person’s perspective through group interaction. Moreover, memories could be triggered by a comment from another participant [32,33]. Otherwise, they can also be triggered by sharing and comparing participants’ own experiences [34].

This study is part of a mixed-method study. The protocol of this mixed-method has been published elsewhere [35]. The medical ethical committee Brabant (METC Brabant) approved the study (project number NL55386.028.15). This study is also registered in the Netherlands Trail Register (number NTR6258). All participants gave written informed consent. Participation was voluntarily and, except for an exit ticket for the parking lot, no financial reward was given.

### Participants and procedure

Eligible patients who experienced an injury, were treated in the shock room of the ETZ Hospital (Elisabeth-TweeSteden Hospital), Tilburg, the Netherlands. These patients were registered in the Brabant trauma registry and a researcher (EV) received a database from this registry. In addition to being treated in the shock room, another inclusion criterion was being aged 18 years or older. Persons were excluded if they had severe TBI (i.e., Glasgow Coma Score ≤ 8), dementia, or insufficient knowledge of the Dutch language (verbal and in writing). Patients’ medical records were reviewed on eligibility. Eligible patients received an information letter and were invited to participate in the study. Then, EV contacted the patients, by telephone, to explain the purpose of the study and to ask for their participation. Patients who were willing to participate in a focus group discussion received additional information about the date, time, and location.

To attain a variety of experiences and a representative sample of the heterogeneous trauma population, patients were divided into three groups: (i) Injury Severity Score (ISS) < 16 (one single injury or mild/moderate injurie(s)), (ii) ISS ≥ 16 (i.e., severe multiple injuries), and (iii) mild or moderate TBI (i.e., Glasgow Coma Score ≥ 9). Six to ten patients were invited to participate in each group. In addition, patients were selected based on sex and age. The researcher (EV) invited equal numbers of male and female patients and a variety of ages for each group in order to attain a variety of experiences and a representative sample of the trauma population. In this way, the presence of maximum variability within the primary data could be warranted, the maximum variation sampling could be clearly set out, and trauma patients with all kind of trauma mechanism and injuries could be included. The purposive sampling method was used [32,33].

In order to obtain reliability and validity [36,37], a manual was developed. The purpose of the focus groups, diversity of study population, and the procedure of the focus groups itself (e.g., introduction by the moderator, questions for participants (e.g., data collection), and finishing the discussion) were set out in this manual. Clear research questions were needed to obtain relevant answers (i.e., validity) and to ensure that the study is replicable (i.e., reliability) [37]. All focus groups had the same structure and were audio-recorded. Two reviewers (EV and BDO) independently reviewed the transcripts to ensure that data saturation (i.e., no new information was found during discussions) was reached. Moreover, to strengthen validity and comprehensiveness, this study was conducted and reported according to the consolidated criteria for reporting qualitative research (COREQ) checklist for qualitative research [36].

### Data collection

The focus group meetings took place in a conference room at the hospital. The focus groups were led by a moderator (EV) and an assistant (MT). The moderator started the focus group by giving an introduction of the moderators and the purpose of the focus group meeting. Then, the patients were asked to share their experiences, by answering the main questions “Which experiences after injury impressed you the most?” and “Can you describe the consequences of injury on your life?””. Then, follow-up questions were asked by the moderator to obtain how these experiences impede or facilitate patients’ wellbeing, for example; “Could you describe your feelings after injury, hospitalization, and rehabilitation?”. In addition, in order to stimulate conversation flow and involve other participants in the discussions, follow-up questions were asked, for instance, “Does someone (i.e. another participant) recognize these experiences, consequences, or feelings?” and “In what way do you experience changes in wellbeing?”. Using this method, the moderator made sure that every participant had the opportunity to interact in the discussion and that participants were motivated to talk with each other [32,36]. Participants’ experiences were clustered on a flipchart on the basis of the trauma procedure; (i) moment of injury, (ii) treatment from medical staff from the ambulance or the trauma helicopter, (iii) treatment in the shock room, (iv) hospital stay, (v) moment of discharge, and (vi) period after discharge and/or rehabilitation. Also, the assistant moderator took field notes, handled logistics, and monitored the audio recording equipment [32].

At the end of each focus group, participants provided information on sociodemographics (i.e., age, sex, marital status, and education level). In addition, they completed the self-report questionnaires; Impact of Event Scale revised (IES-R) for measuring PTSD and the Hospital Anxiety and Depression Scale (HADS) for measuring anxiety and depressive symptoms.

The 22 items IES-R measures symptoms severity of intrusion, avoidance, and hyperarousal. It uses a 5-point Likert scale ranging from 0 (*not at all*) to 4 (*extremely*) [38]. The cut-off score for a probable diagnosis of PTSD is ≥ 33. The IES-R, as well as the Dutch version, has good psychometric properties [38,39].

The HADS assess anxiety (7 items) depressive symptoms (7 items) and uses a 4-point rating scale ranging from 0 (*not at all*) to 3 (*very much*)). Cut-off scores of ≥11 for one of the subscale were regarded as a psychological complaint. The questionnaire is shown to be reliable and valid [40] and has good psychometric properties [41].

### Data analysis

The focus group meetings were analyzed using a phenomenological approach [42]. The recorded focus groups were transcribed verbatim. Then, data analysis proceeded stepwise using the open, axial, and selective coding technique [32,33]. First, open coding was used to identify experiences and consequences of injury on patients’ wellbeing: physical, psychological, and social wellbeing. In addition, moments in time of trauma treatment or recovery, which were related to patients’ experiences were explored. Then, axial and selective coding was used to interpret and explain patients’ experiences by determining different themes and subthemes (level 1 and level 2) based on physical, psychological, and social wellbeing. These codes consisted of short sentences or single words, for example, ‘ASD symptom’ (i.e., theme (level 1) in psychological wellbeing) and ‘nightmares’ (i.e., subtheme (level 2) of ASD in psychological wellbeing), or ‘dependent of care’ (i.e., theme in social wellbeing), ‘loss of control’ (i.e., subtheme level 1 in social wellbeing) and ‘reassurance to hear voice of relative’ (i.e., subtheme level 2 in social wellbeing).

Two researchers (EV and BDO) independently coded and analyzed each of the transcripts Using the computer program Atlas.ti was. Demographics and responses on the questionnaires were analyzed chi-square tests and independent t-tests using SPSS version 24.

## Results

**Fig 1:**
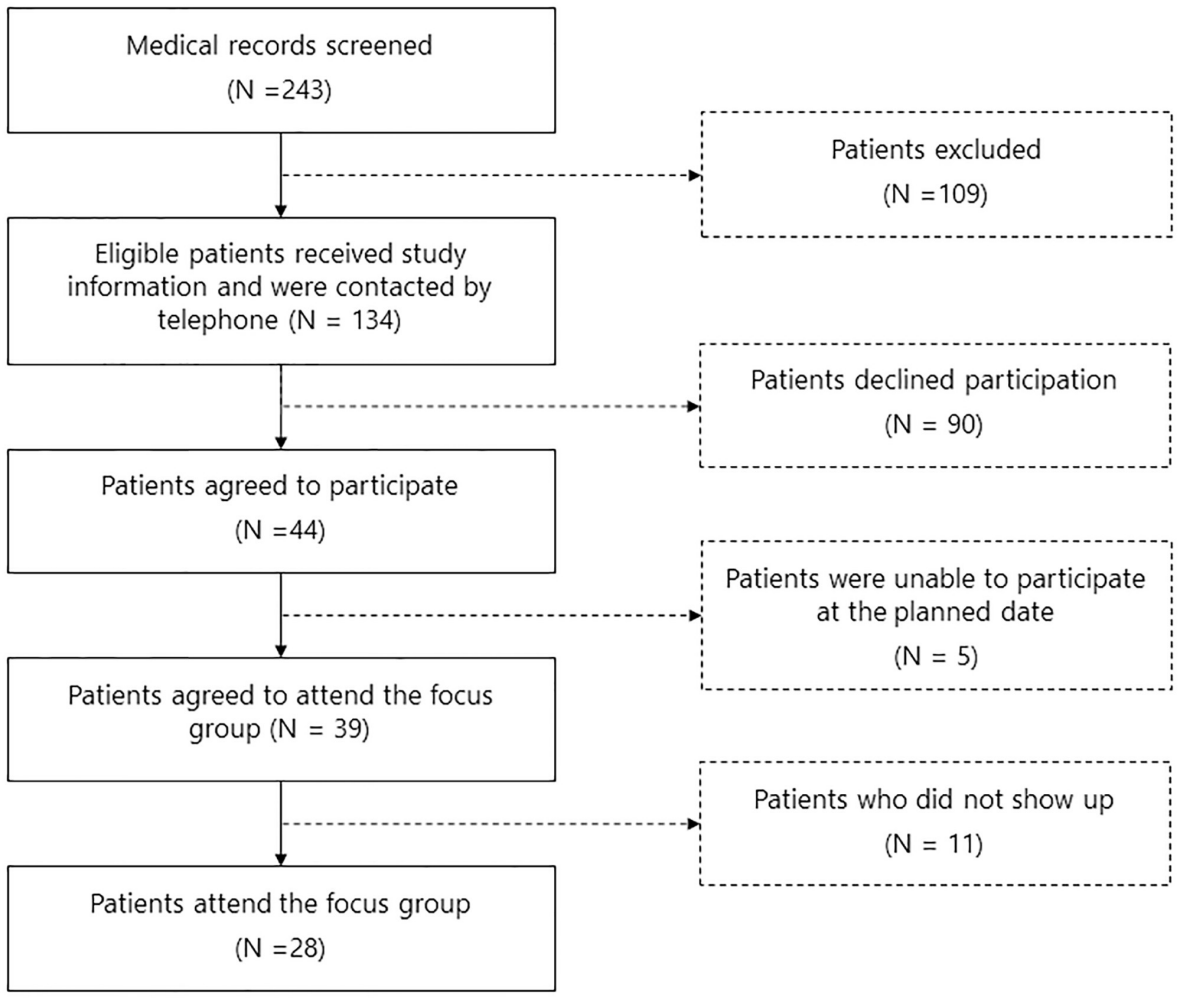
Flowchart of patient inclusion.

After six focus groups data saturation was reached. The duration of the meetings varied between 60 to 90 minutes. In total, 135 patients were invited of which 28 (21%) agreed to participate (Fig 1).

The main reasons for declining participation were that patients indicated that they did not have enough time to participate (22%) or they did not experience any problems after injury (9%). In contrast, a subgroup declined, because participation was too confronting for them (19%). They were afraid that sharing experiences with others could be a trigger for re-experiencing their trauma. The six groups consisted of three up to seven participants (Table 1). The median age was 59.5 (min. 18 –max. 84) and the mean ISS was 11.8 (SD = 9.9).

**Table 1:** Participants’ characteristics.

Age*	58.1 ± 16.1
Sex	
Male (N, %)	20 (71%)
Female (N, %)	8 (29%)
Living situation	
Alone (N, %)	5 (19%)
With parents (N, %)	2 (8%)
With a partner, no children (N, %)	11 (42%)
With a partner and children (N, %)	7 (27%)
Alone, with children (N, %)	1 (4%)
Educational level	
Low (N, %)	4 (15%)
Middle (N, %)	13 (50%)
High (N, %)	9 (35%)
Paid job	
Yes (N, %)	13 (50%)
No (N, %)	13 (50%)
Type of trauma	
Fall (N, %)	18 (64%)
Accident (N, %)	10 (36%)
ISS score *^ǂ^	11.8 ± 9.9
ISS < 16 (N, %)	13 (57%)
ISS ≥ 16 (N, %)	10 (43%)
Time between trauma and focus group (m) *	7.9 ± 3.5
IES score *	21.2 ± 22.0
Avoidance *	0.9 ± 0.8
Intrusion *	1.0 ± 1.2
Hyperarousal *	1.1 ± 1.2
HADS anxiety *	5.8 ± 5.5
No anxiety (N, %)	15 (68%)
Doubtful (N, %)	2 (9%)
Anxiety (N, %)	5 (23%)
HADS depressive symptoms *	5.0 ± 5.3
No symptoms (N, %)	16 (70%)
Doubtful symptoms (N, %)	3 (13%)
Depressive symptoms (N, %)	4 (17%)

* The means and standard deviations are provided, unless stated otherwise.

^ǂ^ ISS scores could be calculated only for patients who were hospitalized after treatment in the shock room and not for patients who were discharged after treatment in the shock room.

*Abbreviations*: ISS = Injury severity score; m = months; IES = Impact of event scale; HADS = hospital anxiety and depression scale.

Based on the IES-R, six (27%) focus group patients had a possible diagnosis of PTSD 12 months after injury. Patients with a possible diagnosis scored different on the subscales. For example, one patient scored *moderately* (score: 2) on avoidance and *extremely* (score: 4) on intrusion and hyper arousal, whereas two other patients scored *quite a bit* (score 3) on all subscales. With regard to the HADS [40], five (22%) patients were anxious and four (17%) had depressive symptoms 12 months after injury. Four patients (17%) showed symptoms of PTSD, anxiety and depression.

During the focus group discussions, seven patients described symptoms of PTSD during rehabilitation, such as having (severe) sleeping problems or nightmares, or re-experiencing trauma. Two of these patients were diagnosed with PTSD by a registered health psychologist, of which one patient (veteran) was diagnosed with PTSD before injury. The other patient developed PTSD as a result of her trauma. This patient also had limited physical (e.g., pain) and psychological functioning (e.g., concentration problems) in such a way that she lost her job and needed to stop her education.

### Physical wellbeing

Table 2 shows the major themes and subthemes of physical wellbeing after injury.

**Table 2:** Major themes and subthemes of physical wellbeing.

Major theme	Subtheme level 1	Subtheme level 2	Moment of procedure
Physical limitations	Inability to communicate	-	Shock room
	No recovery to pre-trauma function	-	Rehabilitation
	Adaptation to physical limitations	Pain, headache or stiffness	Rehabilitation
	Coping	Desire for quick recovery	Rehabilitation
		Intervention by medical staff	Rehabilitation
		Need to slow down	Rehabilitation
Energy level	Activities requires a lot of effort		Rehabilitation

Patients reported not being recovered to the pre-trauma functional level, because physical limitations were still present after 12 months.

“*The physician said that my complaints would diminish over time*. *However*, *I still cannot walk well and I am in pain every day*. *I lost my job and I had to quit my education*. *Most difficult is that I am only 18 years old and I have lost everything (Female*, *ISS < 16)*”.

Patients experienced that the time they needed to recover from activities was much longer than they expected to be. They had to take small steps during rehabilitation, because they experienced physical limitations (e.g., pain or fatigue). Especially severely injured patients (ISS ≥ 16) stated that they ignored physical limitations, because they were motivated to work hard and fully recover as soon as possible.

“*I wanted to recover as quickly as possible*, *but I was hampered by others (rehabilitation specialist or psychotherapists)*. *It was very difficult to cope with that*, *because I wanted to make progress instead of doing nothing (Male*, *ISS < 16)*”.

However, the rehabilitation specialist or physiotherapist often instructed them to slow down in order to respect their physical boundaries. Patients stated that rehabilitation, in this phase, could be frustrating.

“*I had to adapt all the time during rehabilitation*, *because I was not physically capable to rehabilitate the way I hoped and thought I could (Male*, *ISS < 16)*”.

Yet, looking back on this rehabilitation phase, patients acknowledged that the rehabilitation specialist, physiotherapist, and nurses played an important role by guiding the patients how they could recognize, adapt, and cope with their physical boundaries. Moreover, health care professionals (HCPs) educated patients how to balance activities and rest, because activities takes a lot of energy. In this way, patients were able to keep their limitations in mind so they did not cross their boundaries.

“*It takes a lot of effort to do the things I like to do (Female*, *ISS < 16)*”.

### Psychological wellbeing

Table 3 shows the major themes and subthemes related to psychological wellbeing after injury.

**Table 3:** Major themes and subthemes of psychological wellbeing.

Major theme	Subtheme level 1	Subtheme level 2	Moment of procedure
Fear/anxiety	Going to die	-	Injury
	Severe injury	Worse physical outcome	Injury
	Lack of clarity about the cause of trauma	-	Shock room
	No memories	Nightmares	ICU
	Future perspective	-	In hospital
Helplessness	-		Shock room
	Motivation for recovery	-	Rehabilitation
Uncertainty	Lack of clarity about treatment	-	Shock room
	Future perspective	-	In hospital
			Rehabilitation
Processing trauma	Severity of the injury	-	Shock room
	Realizing that one survived	-	In hospital
	Trust in a positive outcome	-	In hospital
	Acceptance	Difficulties with acceptance	Rehabilitation
	Mentally unstable	-	Rehabilitation
Coping	Avoidance	Fear of falling	Rehabilitation
		Facing emotions	Rehabilitation
	Relapse to an old addiction (*e*.*g*., *smoking/drinking*)	-	In hospital Rehabilitation
	Feelings of revenge	-	Rehabilitation
ASD symptoms	Nightmares	-	In hospital (*e*.*g*., *ICU*)
	Flash backs	-	In hospital
PTSD symptoms	Re-experiencing trauma	-	Rehabilitation
	Being mentally unstable	-	Rehabilitation
	Sleeping problems	-	Rehabilitation
Subjective personality changes	Easier satisfied	-	Rehabilitation
	Response shift	-	Rehabilitation
	No memories of personality before trauma	-	Rehabilitation
Emotion changes	Intensified	-	Rehabilitation
Behavioral changes	Being more careful	-	Rehabilitation
Cognitive function	No memories about injury	-	Injury
			Shock room
	Memory difficulties	-	Rehabilitation
	Mental fatigue	-	Rehabilitation
	Forgetful	-	Rehabilitation
	Reduction in information processing speed	-	Rehabilitation
	Difficulties with recognition of persons	-	Rehabilitation
	Concentration difficulties (*e*.*g*., *reading*)		Rehabilitation
	Resumption of work		Rehabilitation

*Abbreviations*: ICU: Intensive care unit; ASD: acute stress disorder; PTSD: Posttraumatic stress disorder

Severely injured patients experienced a fear of dying short after injury, during treatment in the ambulance, and in the shock room.

“*Then just after injury*, *I saw blood spouting from my leg*. *I thought that I had an arterial bleeding and was convinced that I would die within a few minutes (Female*, *ISS ≥ 16)*”.

During hospitalization and recovery, patients realized that they survived the injury. The previously experiences fears, like fear of dying, were followed by a fear for permanent physical limitation.

“*The perspective of ending up in a wheelchair was difficult*, *because I am a fanatic sportsman (Male*, *ISS ≥ 16)*”.

The fear for permanent physical limitations caused uncertainty about the future. Patients did not know what to expect. In addition, patients who were sedated, were unconscious, or had posttraumatic amnesia during treatment in the ambulance and shock room, described that they were confused and anxious about what had actually happened.

“*My anxiety emerged during treatment in the shock room*. *I mainly had questions about the cause of my injury*, *for instance*: *‘What did I experienced*?*’ and ‘What has happened to me’*? *(Male*, *ISS ≥ 16)*”.

“*The most impressive memory was when I woke up on the ICU after three days of being unconscious*. *I thought I had a nightmare*, *but my nightmare was in fact reality (Male*, *ISS ≥ 16)*”.

Then, during hospital stay and after being discharged, patients described symptoms of ASD during hospitalization and/or PTSD during rehabilitation.

“*During the first weeks after injury*, *I had a lot of nightmares about my leg amputation (Female*, *ISS ≥ 16)*”.

“*When I am sad*, *I see the white car approaching me and I re-experience the injury again (Female*, *ISS ≥ 16)*”.

In contrast, patients stated that feelings of helplessness and being dependent of others were difficult experiences to cope with. Especially severely injured patients (ISS ≥ 16) discussed that they were motivation to recover, because they wanted to be autonomous instead of feeling helpless.

“*I did not want to feel helpless*. *Therefore*, *I was very motivated to recover (Male*, *ISS ≥ 16)*”.

In addition to patients’ frustrations, angriness, and other negative feelings, they also stated that they experienced adverse and favorable outcomes concerning their (subjective) personality, emotions, and behavior. Changes in (subjective) personality are describe by the participant selves and not determined by a questionnaire. Patients felt satisfied with these changes.

“*The trauma changed me*. *Before my injury*, *I was quite a reserved person*, *but now I am more open and kind (Male*, *ISS ≥ 16)*”.

“*My emotions became more intense*. *For example*, *when I am happy*, *I am happier than I used to be (Male*, *ISS ≥ 16)*”.

“*Due to trauma*, *I became easier satisfied instead of being a perfectionist (Female*, *ISS < 16)*”.

Patients often had no memories about their injury and treatment in the ambulance. The first memories emerged during treatment in the shock room or during hospitalization. Patients reported mental fatigue during rehabilitation. Moreover, they experienced (in some cases) permanent cognitive problems with recognition of persons, concentration (e.g., reading), reduction in information processing speed, and being forgetful. They also experienced mental fatigue.

“*It just feels like I am ten years older*. *My mental speed is reduced*. *I am not the person who I used be (Male*, *ISS ≥ 16)*”.

Cognitive dysfunction resulted in problems with resumption of work.

“*I would like to have a job*, *however*, *I have to accept that I am not able to work anymore*, *because I am not able to concentrate and cannot even read a book (Male*, *ISS < 16)*”.

To deal with psychological consequences (e.g., anxiety, changes in subjective personality, and cognitive dysfunction, Table 3), some patients described to use an avoidance coping strategy during hospitalization and/or rehabilitation. As they avoided trauma-related physical activities. They had a fear of falling.

“*My bike is still there but I do not look at it anymore (Male*, *ISS < 16)*”.

Patients tended to tone down the impact of their trauma by thinking: ‘It is just an injury’. However, looking back on the trauma procedure, they acknowledged that they should not underestimate the impact of their trauma.

### Social wellbeing

Table 4 shows the major themes and subthemes of social wellbeing after injury, including experiences that are related to the environment.

**Table 4:** Major themes and subthemes of social wellbeing.

Major theme	Subtheme level 1	Subtheme level 2	Moment of procedure
Impact on relatives	Fear that patient would be dead	-	Injury Shock room
	Panic	-	Injury
	Overanxious	-	Rehabilitation
	Relationship problems	-	Rehabilitation
Dependent of care	Loss of control	Reassurance to hear voices of relatives	Injury
Social support	Help from neighbors	-	Rehabilitation
	No one to fall back on	-	Rehabilitation
	Need for social interaction	-	Rehabilitation
Communication health care provider ↔ patient	Reassurance by nurse	Surrender to care	Shock room
	Lack of clarity about injury severity	Need for further explanation	Shock room
	Lack of clarity about patients’ treatment		Shock room
		No time to respond because of treatment protocol	Shock room
	Feel not taken seriously	-	In hospital
	Lack of clarity about future expectations	Need for further explanation	Rehabilitation
Take self-initiative to receive medical care	-	-	In hospital
			Rehabilitation
Communication health care providers → relatives	No update about treatment	-	In hospital
Communication between medical staff	No information transfer	-	In hospital
Communication hospital → GP	No information transfer	-	Rehabilitation
Communication hospital ↔ authorities	No information transfer	-	Rehabilitation
Communication authorities ↔ patient	No information transfer	-	Rehabilitation
Media attention	Negative effect of incorrect information	-	Injury
	Prejudices from others resulting from false information	-	Rehabilitation
Practical problems	Insurance	Financial problems	Rehabilitation
		Claim for damages	

*Abbreviations*: ↔: between; →: from–to; GP: General practitioner.

Patients’ injury had an impact on their family, because their family feared that the patient would not survive the physical trauma.

“*The impact of my trauma is bigger for my family than for myself (Male*, *ISS ≥ 16)*”.

This fear often resulted in partners who became overanxious during rehabilitation.

“*My wife pleases me not to go on the bike by saying*: *“Go find another hobby” (Male*, *ISS < 16)*”.

In addition, a patient acknowledged that his injury, the fact that he became dependent of others had negatively influenced his marriage.

“*I was angry all the time because of physical limitations I became dependent of others*. *It was difficult for my wife to cope with my angriness*. *Due to my rehabilitation*, *I felt a little bit better*, *because limitations decreased (Male*, *ISS < 16)*”.

Patients experienced a loss of control when they had difficulties with being dependent of care from family and health care providers.

“*It was frustrating to be dependent of care (e*.*g*., *need help by taking a bath)*, *because I found it difficult to be naked*, *but I had no choice (Female*, *ISS < 16)*”.

Although being dependent of others can be difficult, patients were grateful with the help they received from others. Moreover, patients thought that support of relatives and friends could help them to recover.

“*When I got out of bed I was not able to walk*. *In a period of time*, *I have learned to walk again step by step with the support of others*. *In the future*, *I will ride my bike again (Male*, *ISS < 16)*”.

Moreover, patients felt reassured when they heard voices of relatives shortly after injury. Especially elderly patients (i.e., > 70 years old), who were dependent of relatives’ care before injury, reported that the need for the right social support is crucial. These patients experienced more difficulties with social support, because they had a limited social network and in some cases (almost) no one to fall back on compared with younger participants.

“*I am all alone after losing my wife a few years ago (Male*, *ISS ≥ 16)*”.

“*I need a lot of help from my neighbors*, *because my children live far away*”.

Almost every participant thought that communication could be improved between medical staff in hospital, general practitioners, authorities, and patients. Since almost every patient provided an example of not being well or incorrectly informed by a HCP. For instance, during hospitalization, patients needed more information about their treatment or prognosis of recovery.

“*If they (physicians) explained the consequences of my brain injury more clearly*, *then I would be more able to cope with the consequences (♂*, *ISS≥16)*”.

Patients illustrated that medical staff could reassure them during treatment. In addition, they could also clarify patients’ injury severity and inform them about their treatment, prognosis, and future outcomes. However, during hospital stay, patients felt that there was limited time for information transfer. Furthermore, they had to take on one’s own initiative for receiving care. Patients thought that good communication could facilitate recovery during hospital stay and recovery.

“*I had to ask everything*, *including my medication*, *because I did not receive the care I needed (Male*, *ISS < 16)*”.

“*I had to wait a while to be referred for rehabilitation*. *So*, *I was the one who arranged physiotherapy during that period*, *because I wanted to recover (Male*, *ISS ≥ 16)*”.

Patients described that lack of clarity about their injury severity and trauma treatment emerged during treatment in the shock room.

“*It (shock room) was very hectic*, *because different physicians were present*. *Also*, *I went back and forth to several rooms for different examinations*. *I had no idea what happened during treatment (Male*, *ISS ≥ 16)*”.

At that moment, patients experienced a lack of communication between themselves and HCPs since there was no time to communicate.

“*One of the medical staff asked me*: *“Can we cut your clothes*?*” But before I could answer*, *I lay in my naked butt (Male*, *ISS < 16)*”.

Patients felt that they were not being taken seriously due to a lack of communication. If information was provided, some patients did not completely understand it. Medical jargon was often used. In addition, multiple physicians were involved in patients’ treatment, but they did not introduce themselves or explained what they were doing. Patients felt a loss of control in this overwhelming situation. Therefore, due to a lack of information transfers, patients reported that being well reassured short after injury and during treatment in the shock room could help them to surrender to medical care.

“*The nurse was very kind to me*. *She told me*: *“It is going to be ok and we will take good care of you*.*” (Female*, *ISS < 16)*”.

Moreover, patients reported miscommunication between authorities (e.g., hospital and general practitioners or hospital and rehabilitation specialists).

“*I assumed that my GP was informed by the hospital about my injury*. *Unfortunately*, *he did not receive any information (Male*, *ISS < 16)*”.

Patients described that the media attention negatively affected patients’ social interactions after injury, because the media provided false information.

“*Within half an hour there was some story on the news about two seriously injured people*, *but that was incorrect*. *This news caused a lot of gossip in town (Male*, *ISS < 16)*”.

After being discharged and during rehabilitation, patients reported having problems with practical issues, such as problems with finance, health insurance, or difficulties with the re-examination for their driver’s license. Although patients were dependent on authorities, they needed to take own initiative to solve these problems.

“*I am frustrated because the claim for damages has been rejected (Male*, *ISS ≥ 16)*”.

## Discussion

This study aimed to explore and describe patients’ experiences and wellbeing after injury, treatment, and rehabilitation. Moreover, factors that impede or facilitate patients’ wellbeing were examined. Patients explained that they did not recovered to their pre-injury functional level up to12 months after injury. One of the reasons could be the presence of PTSD, anxiety, and depressive symptoms 12 months after injury, which is in line with previous studies [28,43]. Moreover, patients experienced feelings of helplessness, a fear of dying, and/or a fear for a worse outcome short after injury and during treatment in the shock room. They illustrated that feelings of loss of control occurred, because treatment in the shock room was explained as overwhelming and patients needed to surrender to care. Also, patients stated that they needed more information about the injury and treatment when they were in the ambulance and shock room, especially when they did not remember their injury. In some cases, it can be difficult to inform the patient when rapid screening and treatment in the shock room is crucial for survival. In this life-threatening phase, the main goal is fast recognition and prompt treatment of severe injuries [10] by ‘treat first what kills first’ (i.e., ABCDE-method in trauma treatment) [44]. This has shown to be essential for long-term outcomes [10]. Nevertheless, patients illustrated that reassurance by a physician or nurse could help them to surrender to medical care. Moreover, in line with other studies, nurses could help them to cope with feelings of insecurity [30,45].

Furthermore, this study showed that patients had to deal with adverse changes in physical (i.e., pain, stiffness), emotional, cognitive functioning [46], and (subjective) personality [47,48]. For instance, memory impairment, loss of autonomy, and problems in work, marriage and income, could play an important role as obstructive indicators for these changes [46]. In line with the literature, changes in personality could be related to TBI [48–50], while patients’ perception on positive changes in (subjective) personality or emotions might be a result from a change in internal standards or values, i.e., response shift [47]. Furthermore, satisfaction with care improved if a health care provider was interested and involved in patients’ care and recovery [28,51]. Especially during rehabilitation, when patients struggled with resumption to work and financial stress, the need for positive support from their employer or authorities was high [26,29,52].

In addition, patients stated that good communication regarding treatment and rehabilitation is imperative and it needs further improvement [28]. Lack of clarity about patients’ treatment or prognosis, emerged when patients were not well, insufficient, or incorrectly informed by the doctor about expectations and consequences of injury on their wellbeing (i.e., physical, psychological, and social). Moreover, patients felt that they were not being heard by HCP. There is a need for further explanation about the outcome of recovery on all domains. One of the reasons for lack of clarity or insufficient information transfer was that patients could not remember the provided information as a result of cognitive deficits from injury. Another reason could be found in limited time to contact between patients and HCPs, which can be a result of high workload and time pressure [53]. Furthermore, patients had to take self-initiative for receiving care (e.g. asking about their own medication), which could be frustrating when they were dependent of others. Miscommunication could be due to a lack of connection or expectations in communication [51]. For example, the content of communication from a trauma surgeon could be oriented on medical or physical outcomes whereas patients’ content was focused on personal (i.e., emotional of psychological) needs [51]. Another reason for the presence of miscommunication could explained by the concept of testimonial injustice (i.e., gaining knowledge by being told by others) [54], which is part of epistemic injustice [55].

To our knowledge, this is the first study that explored patients’ perspectives on injury, treatment in the shock room and hospital, and rehabilitation using a focus group design. This provided knowledge insight which experiences were present on a specific moment after injury. For instance, after being treated in the shock room, a fear of dying during treatment in the shock room could change in anxiety for permanent physical limitations during hospitalization of rehabilitation. Moreover, the focus has been on psychological consequences and functioning. These topics were under evaluated in the field of trauma research. Moreover, trauma patients with different types of injuries (e.g., fractures, upper and/or lower extremity injuries, traumatic amputation, and TBI) and trauma mechanism (motor vehicle accident, fall, and collision) were included. The qualitative design of this study facilitated an in-depth exploration about patients’ experiences. In-depth discussions were stimulated, because participants shared their perspectives. Finally, the focus groups were led by the same moderator and conducted in the same standardized manner. The focus groups were conducted using a reliable and valid methodology which resulted in robust data with group data saturation [32,33,42]. To facilitate validity, all participants were capable to answer the research questions. They also provided a whole range of responses to the research questions to attain reliability.

Nevertheless, some limitations must be taken into account. First, the low response rate (21%) probably implied response bias [56]. In line with the literature [56,57], patients who declined participation were not interested, because they did not have any physical or psychological problems after trauma. Other patients explained that participation was too difficult, because they could be faced with their psychological problems (e.g., re-experiencing the trauma) when they were triggered by the group discussion. They did not want that. Another limitation was that one of the six focus group consisted of only three participants, because two other patients did not show up. Although this small number could influence the quality of the group dynamic [58], all three participants participated in the discussions in a way that group interaction occurred. This is in line with the literature, which illustrate that smaller focus groups could allow participants to open up about their experiences instead of larger groups [59]. Nevertheless, larger groups can facilitate more in-depth exploration of a persons’ perspectives and ideas. Third, selection bias could have occurred, because participants needed to be capable provide informed consent form. Otherwise, without consent, persons could not participate in this study. Our study population consisted of mainly Caucasian participants since sufficient knowledge of the Dutch language was an inclusion criterion.

Results from this qualitative study obtained several implications for future research and clinical practice. Since only patients participated in this study, future research could focus on how trauma care and patients’ recovery can further be improved by studying HCPs’ (e.g., trauma surgeon, emergency doctor, rehabilitation specialist, etc.) perspectives, their expectations and their role in providing health care. In addition, health care providers must be aware that, in addition to medical traumas, patients can suffer from psychological traumas (e.g., ASD and PTSD) and impaired wellbeing directly or months after injury. Nevertheless, HCPs’ contribution in care might affect patients’ recovery, because satisfaction with care could facilitate recovery. In order to predict who is at risk for psychological problems and disorders, patients can be screened almost directly after injury using the Injured Trauma and Survival Screen (ITSS) [60] or the Psychosocial Screening Instrument for physical Trauma patients (PSIT) [61]. Then, patients can be prevented from physical, psychological, and social consequences by providing early psychological treatment during hospitalization to improve patients’ wellbeing [62].

## Conclusion

Patients reported that their injury had an impact on their physical, psychological, and social wellbeing after injury. These consequences were present up to 12 months after injury. HCPs with the knowledge on physical, psychological, and social consequences could, according to patients, improve anticipation on patients’ needs. This might contribute to patients’ satisfaction with health care.
